# Presumed cytomegalovirus retinitis late after kidney transplant

**DOI:** 10.1590/2175-8239-JBN-2020-0254

**Published:** 2021-05-03

**Authors:** Filipa Silva, Klaus Nunes Ficher, Laila Viana, Inês Coelho, Juliana Toniato Rezende, Daniel Wagner, Maria Lúcia Vaz, Renato Foresto, Helio Tedesco Silva, José Medina Pestana

**Affiliations:** 1 Centro Hospitalar do Porto, Departamento de Nefrologia e Transplante Renal, Porto, Portugal.; 2 Universidade Federal de São Paulo, Hospital do Rim, Departamento de Nefrologia, São Paulo, SP, Brasil.; 3 Hospital Amato Lusitano, Departamento de Nefrologia, Castelo Branco, Portugal.; 4 Universidade Federal de São Paulo, Hospital do Rim, Departamento Infeccioso, São Paulo, SP, Brasil.

**Keywords:** Cytomegalovirus Infections, Kidney Transplantation, Immunosuppression, Infecções por Citomegalovirus, Transplante de Rim, Imunossupressão

## Abstract

Cytomegalovirus (CMV) retinitis is a rare manifestation of CMV invasive disease and potentially threatening to vision in immunocompromised individuals. Clinical suspicion is fundamental since it is an unusual entity with a progressive and often asymptomatic installation over a long period. The authors report a 70-year-old man with diabetic nephropathy who underwent a kidney transplant (KT) in August 2014 with good clinical evolution. No previous CMV infection or episodes of acute rejection were reported. Five years after transplant, he was admitted due to a reduced visual acuity of the left eye with seven days of evolution with associated hyperemia, without exudate. The ophthalmologic evaluation was compatible with acute necrosis of the retina and presumed associated with CMV infection. He had a progressive improvement after ganciclovir initiation. CMV retinitis is one of the most serious ocular complications in immune-suppressed individuals and can lead to irreversible blindness, and because of that, early diagnosis and treatment remains crucial in obtaining the best visual prognosis in affected patients. Secondary prophylaxis with ganciclovir is not consensual, neither is the safety of reintroducing the antimetabolite in these cases.

## Resumo

A retinite por citomegalovírus (CMV) é uma manifestação rara de doença invasiva por CMV e potencialmente ameaçadora para a visão em indivíduos imunocomprometidos. A suspeita clínica é fundamental, uma vez que se trata de uma entidade incomum, com uma instalação progressiva e frequentemente assintomática durante um longo período. Os autores relatam um homem de 70 anos de idade com doença renal do diabetes que foi submetido a um transplante renal (KT) em Agosto de 2014 com boa evolução clínica. Nenhuma infecção anterior por CMV ou episódios de rejeição aguda foram relatados. Cinco anos após o transplante, ele foi internado devido a uma acuidade visual reduzida do olho esquerdo com sete dias de evolução com hiperemia associada, sem exsudato. A avaliação oftalmológica foi compatível com a necrose aguda da retina e presumivelmente associada à infecção por CMV. Ele teve uma melhora progressiva após o início do ganciclovir. A retinite por CMV é uma das complicações oculares mais graves em indivíduos imunossuprimidos e pode levar à cegueira irreversível e, por isso, o diagnóstico e o tratamento precoces continuam sendo cruciais para obter o melhor prognóstico visual em pacientes afetados. A profilaxia secundária com ganciclovir não é consensual, tampouco a segurança de reintroduzir o antimetabólito nestes casos.

## Introduction

Cytomegalovirus (CMV) infection remains one of the most common complications affecting solid organ transplant (SOT) recipients, conveying higher risks of complications, graft loss, morbidity, and mortality^1^. Nowadays, due to CMV preventive strategies among SOT recipients, the epidemiology of the disease has changed. In patients receiving prophylaxis, CMV disease occurs after its suspension and not in the first three months after the transplant, as was typical before prophylaxis introduction^1^^,^ 2.

CMV infection is defined as the presence of CMV replication in tissue, blood, or other bodily fluids regardless of symptomatology, while CMV disease requires CMV infection that is accompanied by clinical signs and symptoms^2^. When CMV disease happens after completion of antiviral prophylaxis it is denominated delayed-onset CMV disease, and when it occurs more than 12 months after transplantation, it is classified as late onset^3^.

CMV retinitis is a rare manifestation of CMV invasive disease and potentially threatening to vision in immunocompromised individuals. Clinical suspicion is fundamental since it is an unusual entity with a broad spectrum of clinical presentation varying from asymptomatic to a severe disease complicated with retinal detachment^4^^,^ 5. Due to the often asymptomatic installation, the diagnosis can be delayed over a long period, ranging from nine months to one year depending on the studies^6^.

The aim of our study was to describe a rare case of late CMV disease manifested as retinitis in a kidney transplant recipient.

### Clinical case

A 70-year-old man with chronic kidney disease due to diabetic nephropathy started hemodialysis in June 2013. He underwent a kidney transplant from an expanded criteria deceased donor in August 2014 [kidney donor profile index (KDPI) of 93%; mismatch of one in HLA-A, one in -B and zero in -DR; and calculated panel reactive antibody (cPRA) of 20% in class I and 0% in class II, without donor-specific antibodies (DSA)]. He had positive CMV IgG serology and donor's serology was unknown. He received induction therapy with a single 3mg/kg dose of anti-thymocyte globulin and maintenance therapy with tacrolimus (TAC), mycophenolate sodium (MPS), and prednisone. No CMV prophylaxis was given. He had a favorable clinical course maintaining a serum creatinine of 1.2mg/dL after five years of transplantation (eGFR CKD-EPI 61mL/min/1.73m^2^), without either CMV infection or acute rejection episodes.

One year after the transplant, he was diagnosed with clear cell carcinoma in the left native kidney and underwent curative nephrectomy. At this time, the MPS dose was reduced to 360mg BID and TAC blood concentrations were maintained between 7 to 12ng/mL.

**Figure 1:**
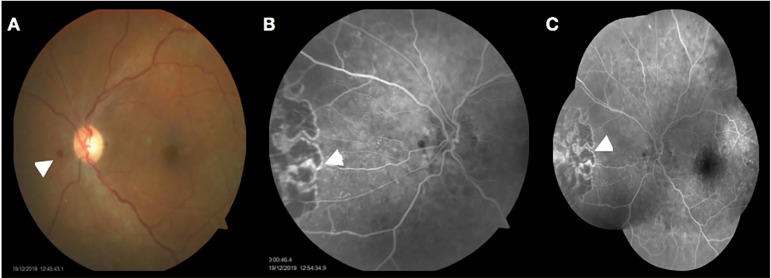
Retinography and angiofluoresceinography performed after several days of treatment with ganciclovir. A - Retinography of the left eye with evidence of intra-retinal hemorrhage (arrowhead). B - Peripheral injury at the level of the nasal retina corresponding to a scar from a previous injury. C - Angiofluoresceinography of the entire fundus. Presence of lesions of diabetic retinopathy and possible retinitis scar at the level of the nasal retina (arrowhead).

He was admitted in December 2019 due to seven days complain of reduced visual acuity of the left eye, associated with local hyperemia, but without any exudate. He had no complaint in the right eye. He underwent a full ophthalmologic evaluation yielding a diagnosis of suspected herpetic-associated retinitis. Empirical treatment with intravenous acyclovir was immediately started and MPS was discontinued. Thorough investigation for other states of immunodeficiency was negative, including negative serologies for HIV, syphilis, bartonellosis, and toxoplasmosis. The blood viral load for *Herpes simplex* and *Varicella Zoster* by polymerase chain reaction (PCR) was undetectable, but was positive for CMV (49 UI/mL - lower limit of quantification is <31 UI/mL). At that time we were unable to perform a quantitative nucleic acid amplification testing (QNAT) using ocular fluids. The hypothesis of tumor recurrence or possible neoplastic lesions was ruled out by computed tomography. Six days later, the patient evolved with progressive worsening of visual acuity, involving also the right eye. A follow-up ophthalmological evaluation revealed upper wedge retinitis with significant arteriolar sheath, micro-hemorrhages, vitreitis, and temporal pigmentary alteration. The antiviral therapy was changed to ganciclovir in order to cover both the possible agents involved, Herpes and CMV. At this time CMV DNAemia was undetectable. There was a progressive improvement in visual complaints, and another ophthalmological evaluation [Figure 1] showed progressive healing of retinal lesions 21 days after the inception of ganciclovir. Treatment was extended for 28 days based on follow up ophthalmological evaluation of complete healing of the retina lesions. He was discharged from the hospital with improvement of his visual acuity, and no secondary prophylaxis was instituted.

## Discussion

The authors described a rare case of a suspected CMV-associated retinitis in a kidney transplant recipient after five years of transplantation, without detection of any concomitant immunodeficiency.

Cytomegalovirus is a ubiquitous virus, member of the *Herpesviridae* family, and presents as an opportunistic infection in 75-85% of cases^7^. While most infections in immunocompetent individuals are benign and self-limited, in immunocompromised patients, it is a clinically significant disease with high morbidity and mortality^1^^,^ 2. CMV disease is considered the single most prevalent complication following solid organ transplantation^8^ and can affect almost every organ, being the gastro-intestinal tract the most frequent organ involved^6^. In the eye, the clinical picture consists of viral necrotizing retinitis and is a sight-threatening organ-invasive manifestation^4^.

When CMV infects a cell, the viral double-stranded deoxyribonucleic acid (DNA) genome migrates to the cell nucleus where the subsequent course of the infection depends upon the state of activation or differentiation of the cell^9^. When latently infected macrophages and dendritic cells become highly activated the virus reactivates and replicates, situation that is normally controlled by the immune system. Despite the presence of anti-CMV antibodies, CD4+ and CD8+ T cells are the most important immune response against CMV, but immune-compromised patients are not capable to generate this response against the virus^9^.

In the eye, active CMV affects primarily the vascular endothelial cells followed by retinal pigment epithelial cells causing viral cytopathic effects and subsequent retinal necrosis, the hallmark of CMV retinitis^10^. Uncontrolled local replication of CMV occurs, causing retinal tissue death and leading to blurred vision, retinal detachment, and ultimately blindness^9^.

Before highly active antiretroviral therapy (HAART) was available, CMV retinitis occurred in 20 to 40% of HIV patients, being the most frequent ocular opportunistic infection in this population and accounting for 90% of HIV related blindness^10^. In contrast, CMV retinitis incidence in other immunosuppressive states is much lower, affecting 1 to 2% of kidney transplant recipients^11^. Nowadays, it became uncommon, only seen in patients with AIDS, but still more common in HIV versus non-HIV immunosuppressed patients^7^. One possible explanation is that CMV and HIV can transactivate each other leading to a greater degree of immunosuppression in HIV patients^12^.

In literature, there are few cases of late retinitis in renal transplant recipients. In 2008, Eid et al^13^ published a retrospective review of all cases of CMV retinitis at the Mayo Clinic. CMV retinitis was diagnosed in 14 eyes of nine patients who received a solid organ or autologous hematopoietic stem cell transplant. Five patients were kidney transplant recipients, and in two of them the disease affected both eyes, at least four years after transplantation. None of them has been documented with CMV viremia. Another report of four cases of CMV retinitis was made by Shimakawa et al^14^. Only one patient, who underwent an ABO-incompatible kidney transplant, had a diagnosis of late disease (3 years and 10 months after the transplant). He had bilateral ocular involvement with positive CMV DNA in aqueous humor, but not with CMV antigenemia.

Clinically, CMV retinitis often manifests with blurred vision, flashing lights, scotomas, and floaters^10^. Typically, CMV retinitis starts as unilateral disease initiating in the midperiphery area and eventually spreading to other areas^10^vision-threatening disease that primarily affects immunosuppressed patients. CMV is the most common ocular opportunistic infection in human immunodeficiency virus (HIV If the diagnosis is made at an advanced stage of the disease, the symptoms tend to be more severe, and the presentation includes severe retinitis, early involvement of the macula, or possible infiltration of the optic nerve^15^^,^ 16. There are two specific patterns of CMV retinitis: granular and hemorrhagic. In the first one there is a brushfire along a line and the second presents centrally with broad geographic zones, satellite lesions, and various levels of hemorrhaging^7^. In our patients, the hemorrhagic pattern predominated. The extent of the lesion tends to be wider with higher degree of immunosuppression^17^. With the progression of the disease, the retina becomes atrophic and the likelihood of retinal detachment increases 10.

Post-transplantation CMV retinitis is usually diagnosed later compared with other organ-invasive manifestations of CMV disease. In 66% of the cases it occurs in the first year after transplantation. Interestingly, early onset CMV retinitis appears to be a component of the systemic, and at times multiorgan, invasive CMV disease, often accompanied by viremia. In contrast, CMV retinitis that occurs in a later period after transplantation is likely to be an isolated target organ disease, in most cases without association of other organs^4^.

CMV retinitis diagnosis is based on clinical and ophthalmologic examination^1^. A positive QNAT in aqueous or vitreous ocular fluid can confirm the diagnosis and may be helpful in cases with atypical clinical presentations. It may be positive before and at the time of diagnosis^1^ and can help to distinguish from other causes of retinitis, such as *herpes simplex, varicella zoster*, or *toxoplasma gondii* 10. Guidelines strongly recommend histology coupled with immunohistochemistry for CMV for the diagnosis of tissue-invasive disease^1^. Herpes virus retinitis cannot be excluded since it is epidemiologically more frequent than CMV retinitis and would also improve under ganciclovir therapy. However, in the presence of ocular features suggestive of CMV infection, active CMV replication, and clinical worsening with acyclovir, the presumptive diagnosis of CMV retinitis is made. Invasive CMV disease is often associated with negative viremia, and positive tissue PCR of the affected organ is required to confirm the diagnosis. The most expressive examples of these situations are retinitis and, more often, gastro-intestinal disease. The possible explanation for this to happen is viral load below the lower limit of detection by currently employed molecular methods or by the different CMV glycoprotein B genotypes that could be associated with viral tropism for specific organs. In this case, CMV documentation in vitreous fluid by NAT would be essential, but, unfortunately, it is not available at our center. Thus, in the presence of eye lesions suggestive of CMV, although not typical, clinical worsening under acyclovir, and previous positive PCR for CMV, we switched our patinent's therapy to ganciclovir with coverage of this agent.

The treatment algorithm is determined for the location and extension of the lesions. When sight-threatening lesions are present, intravitreal injections associated with systemic therapy are recommended. In the absence of immediate sight-threating lesion, the option for systemic therapy alone is reasonable^10^. Currently, there are several drugs available with different routes of administration, like valganciclovir, ganciclovir, foscarnet, and cidofovir. Furthermore no study shows the superiority of one drug over another, and valganciclovir and ganciclovir are generally the first-line agents used, but the choice depends on cost of medication, oral route availability, associated comorbidities, use of potential interfering medication, and predicted compliance to therapy^12^^,^ 18 ^,^ 19.

The duration of treatment is not established but some studies suggest that it can be stopped with security when there are no signs of CMV activity on ophthalmologic evaluation^12^^,^ 20. In the present case, only after all the lesions were healed, the therapy with ganciclovir was stopped.

CMV retinitis frequently recurs after treatment. Iu et al found a recurrence rate of 33.3% after therapy discontinuation and another study found no reactivation after discontinued therapy in 56% of the patients^11^. Reduction of immunosuppression should be strongly considered in patients with severe CMV disease especially those with very high viral loads and refractory disease^12^.

The overall clinical course and visual prognosis of CMV retinitis is similar in non-HIV and HIV patients^8^^,^ 21. Despite advances in anti-viral treatment, the visual prognosis remains poor and is associated with retinal detachment, macular involvement, and poor general health of the patients^21^. In this patient, although there was an improvement in visual acuity during treatment, it was not fully recovered. There was no further complication associated with retinitis.

## Conclusion

CMV retinitis is a serious ocular complication in immunosuppressed individuals and can lead to irreversible blindness. Early diagnosis and treatment remains crucial in obtaining the best visual prognosis in affected patients. The main differential diagnosis is *Herpes simplex* retinitis but other etiologies like *varicella zoster* or *Toxoplasma gondii* should be kept in mind.

The report of this case is essential for bringing awareness on the early recognition and prompt outset of treatment, even without a definitive diagnosis, as it can prevent a severe and irreversible condition. Furthermore, it is not clear when we should reintroduce the antimetabolic with security as well which cases will necessity of secondary prophylaxis.

## References

[1] Kotton CN, Kumar D, Caliendo AM (2018). The Third International Consensus Guidelines on the Management of Cytomegalovirus in Solid-organ Transplantation. Transplantation.

[2] Razonable RR, Humar A (2019). Cytomegalovirus in solid organ transplant recipients-Guidelines of the American Society of Transplantation Infectious Diseases Community of Practice. Clin Transplant.

[3] Khan SF, Yong MK, Slavin MA, Hughes P, Sasadeusz J (2020). Very late-onset cytomegalovirus disease with ganciclovir resistance >15 years following renal transplantation. Transpl Infect Dis.

[4] Eid AJ, Bakri SJ, Kijpittayarit S, Razonable RR (2008). Clinical features and outcomes of cytomegalovirus retinitis after transplantation. Transplant Infect Dis.

[5] Crippa F, Corey L, Chuang EL, Sale G, Boeckh M (2001). Virological, Clinical, and Ophthalmologic Features of Cytomegalovirus Retinitis after Hematopoietic Stem Cell Transplantation. Clinical Infectious Diseases.

[6] Razonable RR, Paya CV (2004). Valganciclovir for the prevention and treatment of cytomegalovirus disease in immunocompromised hosts. Expert Review of Anti-infective Therapy.

[7] Sanghera NK, Newman TL (2010). Cytomegaloviral retinitis from chronic immunosuppression following solid organ transplant surgery: Cytomegaloviral retinitis after organ transplant. Clinical and Experimental Optometry.

[8] Razonable RR, Emery VC, 11th Annual Meeting of the IHMF (International Herpes Management Forum) (2004). Management of CMV infection and disease in transplant patients. 27-29 February 2004. Herpes.

[9] Carmichael A (2012). Cytomegalovirus and the eye. Eye.

[10] Munro M, Yadavalli T, Fonteh C, Arfeen S, Lobo-Chan A-M (2019). Cytomegalovirus Retinitis in HIV and Non-HIV Individuals. Microorganisms.

[11] Kuo IC, Kempen JH, Dunn JP, Vogelsang G, Jabs DA (2004). Clinical characteristics and outcomes of cytomegalovirus retinitis in persons without human immunodeficiency virus infection. American Journal of Ophthalmology.

[12] Jabs DA, Ahuja A, Van Natta M, Dunn JP, Yeh S (2013). Comparison of Treatment Regimens for Cytomegalovirus Retinitis in Patients with AIDS in the Era of Highly Active Antiretroviral Therapy. Ophthalmology.

[13] Eid AJ, Bakri SJ, Kijpittayarit S, Razonable RR (2008). Clinical features and outcomes of cytomegalovirus retinitis after transplantation. Transplant Infect Dis.

[14] Shimakawa M, Kono C, Nagai T, Hori S, Tanabe K, Toma H (2002). CMV retinitis after renal transplantation. Transplantation Proceedings.

[15] Kuppermann BD, Petty JG, Richman DD (1993). Correlation Between CD4+ Counts and Prevalence of Cytomegalovirus Retinitis and Human Immunodeficiency Virus--related Noninfectious Retinal Vasculopathy in Patients With Acquired Immunodeficiency Syndrome. American Journal of Ophthalmology.

[16] Wei LL, Park SS, Skiest DJ (2002). Prevalence of visual symptoms among patients with newly diagnosed cytomegalovirus retinitis:. Retina.

[17] Geng S, Ye J, Zhao J, Li T, Han Y (2011). Cytomegalovirus retinitis associated with acquired immunodeficiency syndrome. Chin Med J.

[18] Stewart MW (2010). Optimal management of cytomegalovirus retinitis in patients with AIDS. Clin Ophthalmol.

[19] Martin DF, Sierra-Madero J, Walmsley S (2002). A Controlled Trial of Valganciclovir as Induction Therapy for Cytomegalovirus Retinitis. N Engl J Med.

[20] Iu LP, Fan MC, Lau JK, Chan TS, Kwong Y-L, Wong IY (2016). Long-term Follow-up of Cytomegalovirus Retinitis in Non-HIV Immunocompromised Patients: Clinical Features and Visual Prognosis. American Journal of Ophthalmology.

[21] Kim DY, Jo J, Joe SG, Kim J-G, Yoon YH, Lee JY (2017). Comparison of visual prognosis and clinical features of cytomegalovirus retinitis in hiv and non-hiv patients. Retina.

